# Marketing Strategies to Encourage Rural Residents of High-Obesity Counties to Buy Fruits and Vegetables in Grocery Stores

**DOI:** 10.5888/pcd14.170109

**Published:** 2017-10-12

**Authors:** Emily Liu, Tammy Stephenson, Jessica Houlihan, Alison Gustafson

**Affiliations:** 1 Department of Dietetics and Human Nutrition, University of Kentucky, Lexington, Kentucky

## Abstract

**Introduction:**

Obesity rates in Appalachia are among the highest in the United States, and knowledge of upstream approaches to decrease prevalence among this vulnerable population is limited. The primary aim of this study was to examine the association between healthy, diet-based, social marketing interventions in grocery stores and frequency of fruit and vegetable intake.

**Methods:**

A social marketing campaign was conducted among 17 grocery stores (N = 240 participant surveys) over 4 months in 5 rural Kentucky counties. Interventions included providing food samples, recipe cards, and promotional discounts on fruits and vegetables and moving high-calorie foods to side aisles.

**Results:**

Most survey participants reported that recipe cards influenced their desire to purchase ingredients as well as fruits and vegetables in general. Results indicated a significant association between the influence of recipe cards and frequency of fruit and vegetable consumption.

**Conclusion:**

Small-scale interventions in grocery stores influenced purchasing choices among Appalachian residents. Working with various store managers and food venues in rural high-obesity communities is a promising way to encourage purchasing of fruits and vegetables.

**Editor’s Note:** This article is the winner of *PCD*‘s 2017 Student Research Paper Contest in the High School category.

## Introduction

Rural residents of the United States have higher levels of adult obesity than do other US residents (1), and Appalachia residents are at a 33% higher risk of diabetes (2). In part because of concern about these health disparities in the region, community-based studies have started focusing on different facets to combat the problems, with diet and shopping behavior being a major area of focus (3).

Given the high percentage of people who report shopping at supermarkets or grocery stores as their primary food stores (60%–85%) (4), grocery stores can provide an avenue for improving shopping choices, such as purchasing more fruits and vegetables, low-fat dairy products, and other healthy items. However, grocery stores, supermarkets, and supercenters also provide avenues for purchasing non–nutrient dense items, such as sugar-sweetened beverages, chips, baked goods, and other processed foods (5). However, provided that shoppers are encouraged to purchase healthy items in a store, stores have potential for improving the nutritional quality of what shoppers purchase and consume (6), thereby improving dietary intake.

Policies have been established to reduce prices on fruits and vegetables and relocating food in stores, and they are correlated with more fruit and vegetable purchases (7). Studies have been conducted on the efficacy of grocery store marketing features on food purchases, with varying degrees of apparent success. One study showed that of the many grocery store features, recipe samples and discount promotions resulted in frequent shoppers being more motivated to purchase healthier foods (8). Another study showed that increasing the perceived access of fruits and vegetables, specifically by focusing on display space, price, variety, and freshness, was associated with an increase in shoppers’ consumption of fresh produce (9). It remains unclear what strategy may be most cost-effective and effective at promoting behavior change; perhaps a combination of all these strategies would be most effective.

The aims of this study were to test the effectiveness of marketing strategies (price reductions, recipe cards and samples, and product placements) on awareness of strategies and in increasing purchasing of fruits and vegetables. This study used a cross-sectional survey of neighborhood grocery stores in 5 counties in rural Appalachian Kentucky with high prevalence of obesity.

## Methods

Grocery stores from the 5 counties participating in this project agreed to take part in the social marketing campaign strategies in stores as a way to improve food-purchasing choices among residents. The 5 counties met eligibility criteria for funding from the Centers for Disease Control and Prevention Obesity Prevention program: having a 40% or higher obesity rates among adults, being a rural geographic area, and being demographically composed primarily of white or Caucasian residents (97%). The reason behind these criteria was to study the rural high-prevalence obese population of Appalachian Kentucky.

Cooperative Extension agents in all 5 counties contacted all grocery store managers in each of their counties and managers of large supercenters in adjacent counties. Grocery store managers were given a letter explaining the social marketing campaign and were offered $100 per store event to offset any costs they might incur by moving food and displays to other locations in the store. Of the 30 stores contacted, 17 agreed to participate in the program. The grocery stores agreed to promote the campaign to their patrons, move merchandise around to promote the foods being sampled, display fruits and vegetables at the front of the store, display Plate It Up Kentucky Proud (PIUKP) materials, and offer a discount on the fruit or vegetable being tasted during the recipe sample.

The PIUKP program was conducted in 17 grocery stores (midsize grocery store with 5–7 checkout counters) or supercenters (large grocery store selling multiple produces and 15 or more checkout counters) during April and May of 2016 and then again in September and October of 2016. PIUKP is a partnership project between the University of Kentucky Cooperative Extension Service, the Kentucky Department of Agriculture, and the University of Kentucky School of Human Environmental Sciences. This project uses undergraduate students to develop and test new recipes using local and seasonal fruits and vegetables. After recipes have been tested, Cooperative Extension agents provide recipe cards, samples, and food demonstrations in their communities as a way to promote consumption. The events were held on various days of the week and various hours to capture a variety of store shoppers. The campaign consisted of displays with the PIUKP banner and recipe samples with recipe cards. Additionally, storeowners moved food that is typically higher in calories away from the front of the store and showcased the fruit or vegetable that was in the recipe. They also moved sugar-sweetened beverages or other “grab and go” items such as chips to a side aisle. Lastly, the storeowner agreed to offer a discount on the fruit or vegetable being sampled with the discount varying from 10% to 15% less than the original price. Each shopper was given a tote bag or gel-pack with the logo if they sampled a recipe. During these 2 months, the program was promoted through advertisements in local newspapers, flyers distributed in schools, and radio announcements.

A customer intercept survey was developed to capture the effectiveness of the in-store marketing events. The survey questions were previously tested for test–re-test among farmers market patrons in rural towns (10). During the marketing campaign, the Cooperative Extension agent or an assistant gave the survey to store patrons who took a recipe card or a recipe sample. A total of 240 surveys were collected from all 5 counties. The survey was conducted at the beginning of the social marketing campaign in April and again in September and October.

STATA version 12 (StataCorp LP) was used to perform statistical analyses. Descriptive statistics and multiple logistic regressions were used to assess the association between the influence of recipe cards and samples with the consumption of fruits and vegetables. All models were adjusted for age, education, and participation in the Supplemental Nutrition Assistance Program (SNAP).

## Results

Mean age of participants was 51 years, and most participants (N = 240) were white (97%) and female (88%) (Table 1). Fifty-eight percent of participants reported a yearly income of less than $40,000, and 19% of participants reported receiving SNAP benefits.

**Table 1:** Characteristics of Participants (N = 240) in Plate It Up Social Marketing Campaign at Grocery Stores in Rural Appalachian Kentucky, 2016

Characteristic	Value^a^
Language is English	100
Female sex	88
Mean age, y	51
Own car	91
Highest grade completed (high school/GED)	34
SNAP recipient	19
White race	97
Income <$40,000	58
Answered yes to the question, “Does having recipe cards available at the market influence your buying of fruits and vegetables while at the market?”	39
Answered yes to the question, “Did the recipe sample available contribute to your buying the ingredients for the recipe you sampled?”	49
Heard of Plate it Up Kentucky Proud	44
Answered yes to the question, “If you took a food sample from the Plate It Up Kentucky Proud Program, did that sample make you want to prepare the food item at home?”	69
Consumption of fruit once per week or less	32
Consumption of vegetables once per week or less	29

Abbreviations: GED, general equivalency diploma; SNAP, Supplemental Nutrition Assistance Program.

a Values are percentages unless otherwise noted.

Only 44% of participants had previously heard of the PIUKP Program. Forty-nine percent reported that the recipe cards influenced the purchasing of ingredients from the recipe, and 39% indicated that recipe cards influenced purchasing fruits and vegetables in general.

When assessing the intervention effectiveness on dietary intake (Table 2), results indicated a significant association between the influence of recipe cards and frequency of fruit and vegetable consumption. Participants who reported that a recipe card influenced the purchase of ingredients related to the fruit and vegetable sample were 2.86 times (*P* = .04; 95% confidence interval [CI], 1.03–7.94) as likely to consume fruit 2 to 3 times per week than were those who reported that the cards had no influence. Participants who reported that the recipe card influenced fruit and vegetable purchases in general were 11.06 times as likely (95% CI; 3.35–36.51;* P* < .001) to consume fruit 2 to 3 times per week and 3.89 times as likely (*P* = .006; 95% CI, 1.46–10.33) to consume fruit at least once per day or more than were those that reported a recipe card not influencing fruit and vegetable purchases.

**Table 2:** Efficacy of Recipe Cards Intervention on Reported Frequency of Fruit and Vegetable Consumption, Rural Appalachian Kentucky, 2016

Characteristic	**Recipe Cards Influenced Purchase of Included Ingredients**		**Recipe Cards Influenced Fruit and Vegetable Purchases in General**	
	OR (95% CI)	*P* Value	OR (95% CI)	*P* Value
**Fruit frequency**				
2 or 3 times per week	2.86 (1.03–7.94)	.04	11.06 (3.35–36.51)	<.001
Once per day or more	1.48 (0.62–3.49)	.38	3.89 (1.46–10.33)	.006
**Vegetable frequency**				
2 or 3 times per week	2.8 (1.08–7.27)	.03	1.65 (0.61–4.45)	.32
Once per day or more	2.32 (0.99–1.04)	.07	2.63 (0.97–7.16)	.06

Abbreviations: CI, confidence interval; OR, odds ratio.

## Discussion

Results of PIUKP grocery store marketing efforts suggest that there is a notable association between consumers’ use of recipe cards and their dietary habits in geographically isolated rural areas of Appalachian Kentucky. Other research indicates that many grocery store features, such as recipe samples and discount promotions, resulted in frequent shoppers being more motivated to purchase healthier foods (8). Our results support these findings, particularly with regard to recipe cards. Our results also indicate how community-based efforts with cooperation from grocery store managers can influence patrons’ purchasing habits. A strength of this study was that these efforts were conducted across various store types. Working with managers from different types of food stores operating in rural communities is a promising strategy for improving purchasing of fruits and vegetables (11).

Fruit and vegetable consumption can reduce a person’s risk of becoming obese (12), and findings from this study can promote healthy shopping behaviors and improve personal diet. Providing opportunities for shoppers to sample different recipes as well as improving the consumer food environment may be a sustainable program approach year-round (13). Store managers who are willing to improve their stores in rural communities can make changes in their stores and thus help to improve fruit and vegetable intake among their customers (14).

Limitations of our study were the cross-sectional survey design, the small sample size, and the lack of causality. More research and different types of interventions, including those that are multipronged or not diet-focused (15), may be needed for more conclusive results on the effects of a social marketing campaign and on an overall look at dietary and shopping behavior among rural residents. Nonetheless, our study results suggest that the implementation of social marketing strategies in rural grocery stores may increase healthy food consumption habits among community residents and should be continued.
